# Micro Characterization of Hot-Rolled Plate of Nb-Bearing Grain-Oriented Silicon Steel

**DOI:** 10.3390/ma15020429

**Published:** 2022-01-07

**Authors:** Yong Wang, Guangqiang Li, Chengyi Zhu, Xinbin Liu, Yulong Liu, Yang Gao, Yu Liu

**Affiliations:** 1 Department of Mechanical and Engineering, Hunan Institute of Technology, Hengyang 421002, China; wangyong@hnit.edu.cn (Y.W.); liuxinbin@hnit.edu.cn (X.L.); 2 The State Key Laboratory of Refractories and Metallurgy, Wuhan University of Science and Technology, Wuhan 430081, China; liguangqiang@wust.edu.cn (G.L.); liuyulong@wust.edu.cn (Y.L.); e84921@baosteel.com (Y.G.); liuyu629@wust.edu.cn (Y.L.); 3 Key Laboratory for Ferrous Metallurgy and Resources Utilization of Ministry of Education, Wuhan University of Science and Technology, Wuhan 430081, China

**Keywords:** hot-rolled grain-oriented silicon steel, Nb(C, N), precipitates, microstructure, texture

## Abstract

In this study, niobium was added into grain-oriented silicon steels, four Nb-bearing hot-rolled bands with Nb content range from 0–0.025 wt% were prepared and a detailed study of the micro characterization (microstructure, texture and precipitates) of hot-rolled bands was carried out by various analysis methods, such as EBSD and TEM. The results indicate that the precipitates in Nb-free steel are MnS and AlN; however, in the Nb-bearing steel they are MnS, AlN and Nb(C, N). The precipitates are finer and more dispersed in Nb-bearing steel, and a stronger pining force was obtained, which contributes to the finer microstructure and less recrystallization fractions of the hot-rolled bands. A larger volume fraction and stronger intensity of Goss texture is presented in steel with 0.025 wt% Nb due to the effective inhibiting effect. However, it has little effect on the changes of microstructure and texture when the Nb content is more than 0.009 wt%.

## 1. Introduction

It is well known that grain-oriented silicon steel has been widely used in electronic devices and electricity, which is mainly made as an iron core for transformers because of its low core loss and high magnetic permeability [1]. Its superior magnetic properties are related to the sharpness of Goss texture ({110}<001>) [2,3], while the inhibitors make significant influences on the formation of the {110}<001> texture [4,5,6,7]. The manufacturing technology of grain-oriented silicon steel should be adjusted according to its composition and types of inhibitors [8]. It is well accepted that the slabs are usually reheated to exceed 1350 °C to ensure that the larger sized inhibitors dissolve completely and re-precipitate with a smaller size; more disperse during the processes of slab reheating and hot rolling [9], which causes a great waste of energy before cold rolling. Therefore, new types of inhibitors with low solution temperatures and strong inhibiting effects should be developed, which will ensure that the reheating temperature of casting billet is relatively low.

Nowadays, many types of inhibitors have been investigated for improving the Goss texture sharpness in grain-oriented silicon steel, such as AlN [10], MnS [11], MnSe [12] and Cu_2−x_S [13]. Niobium forms Nb(C, N) easily in steel, which has a low solution temperature and possesses general properties as the inhibitor of grain-oriented silicon steel. It is added into grain-oriented silicon steels as an inhibitor formation element. It has been reported that lower core losses and higher volume fraction of Goss texture were obtained in steel with Nb(C, N) as inhibitors rather than steel using AlN or MnS as inhibitors [14]. Feng et al. [15,16] reported that Nb(C, N) can be found when niobium is added into steel, which effectively prevents the migration of the grain boundary; this could decrease the reheating temperature, however, the excessive Nb addition creates a negative effect because the dissolved temperature becomes higher with the higher Nb content, which makes it difficult for Nb(C, N) to completely dissolve into the matrix during the reheating process. Furthermore, it has been confirmed [17] that a proper Nb addition into grain-oriented silicon steel is beneficial for the complete abnormal growth of the Goss texture during the secondary recrystallization annealing, which contributes to superior magnetic permeability (*B*_8_ was 1.88 T). N.H. Van Dijk et al. [18] demonstrated that Nb(C, N) particles are small-sized within 10 nm, while the particles with MnS precipitates are large-sized particles in high-strength low alloy steel. It is reasonable to deduce that the size of the Nb(C, N) precipitates accords with the general properties of the inhibitors for grain-oriented silicon steel; thus, a detailed investigation on the characteristics of Nb(C, N) precipitates should be carried out. Our previous studies [19] demonstrate that silicon steel with 0.028% Nb possesses finer and more dispersed precipitates, which shows a stronger pinning force during the entire heat treatment process, resulting in finer microstructure and higher intensity of Goss texture. However, in our previous study, the Al_s_ content was rarely low, which deteriorated the magnetic properties, and a low magnetic induction was obtained. In this paper, the Al_s_ content in silicon steel increases, indicating better magnetic properties. As is known to all, the production of grain-oriented silicon steel includes a series of metallurgic processes, including slab reheating, hot rolling, normalization, cold rolling, decarburization annealing, secondary recrystallization annealing, and so on [20]. The magnetic properties of the finished products may be affected by the precipitates, microstructure, and texture of the steels in any of the above processes. It is essential to study the precipitates, microstructure and texture of each stage in detail. The primary recrystallization microstructure, texture and precipitates were investigated in our previous studies [21,22]. It was reported that the Goss texture originates in the hot rolling stage, and the inheritance of Goss texture plays an important role on the nucleation of {110}<001> secondary grains by way of structure memory [23]. Thus, the characterization of inhibitors, microstructure and texture in the hot-rolled band of grain-oriented silicon steel should be studied in detail.

In a word, the effect of Nb content on the characterization of microstructure, texture and precipitates in hot-rolled grain-oriented silicon steel should be investigated in detail, and four Nb-bearing slabs were prepared in the present work. The purpose of this work is to provide fundamental knowledge for the production of Nb-bearing grain-oriented silicon steel.

## 2. Materials and Experimental Procedure

Silicon steels of compositions within the ranges given in Table 1 were prepared by a vacuum induction furnace and were forged to 210 mm × 120 mm × (30–50) mm of square ingot. The four silicon steels were produced by an identical route. The ingots were reheated at 1180 °C for 90 min, and then were hot rolled to a 2.3 mm thickness. The start and finish rolling temperature were 1000 °C and 940 °C, respectively.

It has been studied that the inhibitors with sizes between 20–100 nm can effectively inhibit the migration of the grain boundary, which presents obvious restraining effects [24]; therefore, only the precipitates within 100 nm were investigated. In this study, the precipitates were observed by JEM-2100 transmission electron microscopy (JOEL, Tokyo, Japan) (TEM) equipped with energy dispersive X-ray spectroscopy (EDS). It was prepared by the carbon extraction replica technique. In order to ensure the rationality and validity of the data, one hundred consecutive TEM images at ×20,000 magnifications were taken for each specimen, and the number density and size of the precipitates was measured by the Image-Pro Plus image analysis software.

The microstructure and texture characterization along the longitudinal section (RD-ND) of hot-rolled bands was carried out in field emission scanning electron microscope (SEM, Apreo S Hivac, Hillsboro, OR, USA) equipped with an electron backscattered diffraction (EBSD) system. The samples for EBSD analysis were prepared by vibration polishing. The EBSD data were analyzed by an HKL Channel 5 EBSD software (Oxford Instruments, Oxford, UK) and presented in the form of orientation map and orientation distribution functions (ODF) in φ_2_ = 45° section.

## 3. Results

### 3.1. Characteristics of the Inhibitors in the Steels

**Figure 1:**
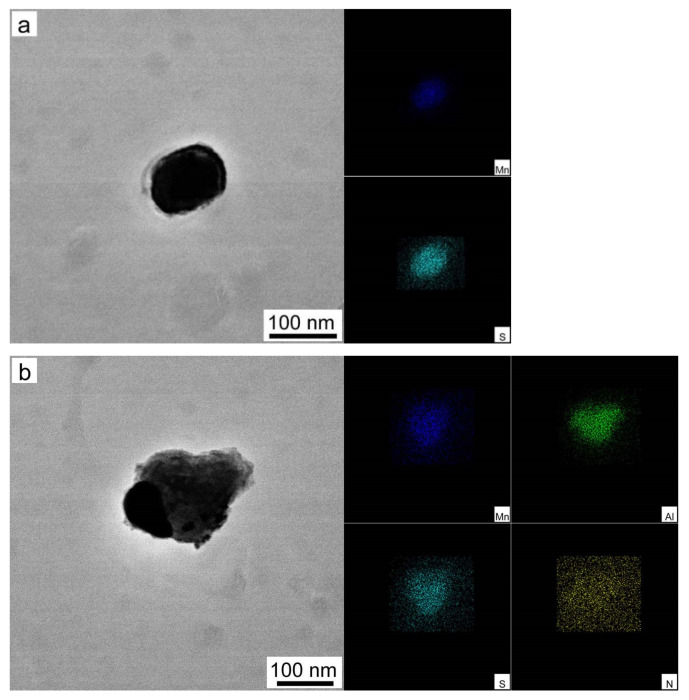

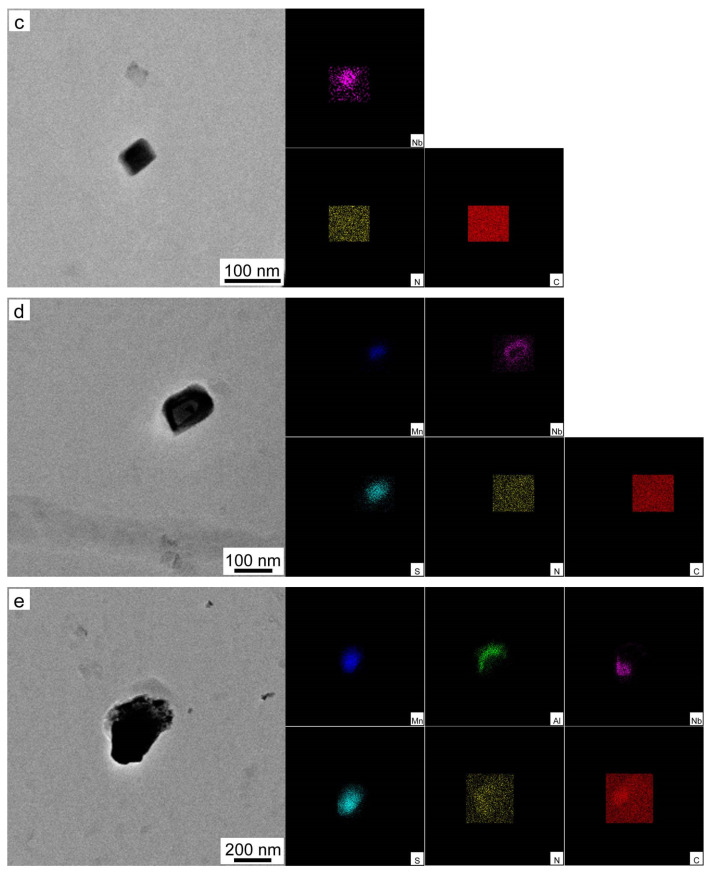
Precipitate morphologies observed by TEM and EDS elemental mapping of precipitates: (**a**) MnS, (**b**) MnS + AlN, (**c**) Nb(C, N), (**d**) MnS + Nb(C, N), and (**e**) MnS + Nb(C, N) + AlN.

Figure 1 shows the TEM micrographs of particles of hot-rolled bands and MnS, and composite precipitates of MnS and AlN are found in specimen S1. It can be observed that MnS is spherical and the composite precipitate of MnS and AlN is nearly ellipsoidal-shaped. However, not only MnS and composite precipitates of MnS and AlN are found in hot-rolled bands of Nb-bearing grain-oriented silicon steel, but also rectangular Nb(C, N), composite precipitates of MnS and Nb(C, N) and composite precipitates of MnS, Nb(C, N) and AlN are observed, as shown in Figure 1c–e. It can be observed that the distribution of carbon in EDS elemental mapping analysis is not obvious, because the precipitate observation specimens were prepared by carbon extraction replica technology. The distribution of carbon in EDS elemental mapping analysis is affected by the carbon film.

**Figure 2:**
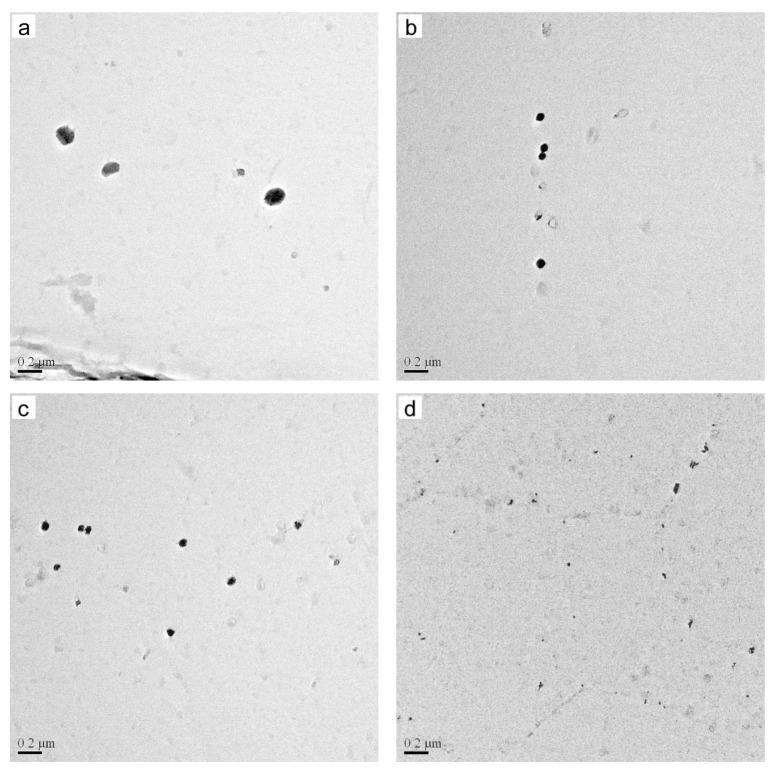
Typical distribution of precipitates in four samples: (**a**) Nb-free, (**b**) Nb-0.005 wt%, (**c**) Nb-0.009 wt% and (**d**) Nb-0.025 wt%.

**Figure 3:**
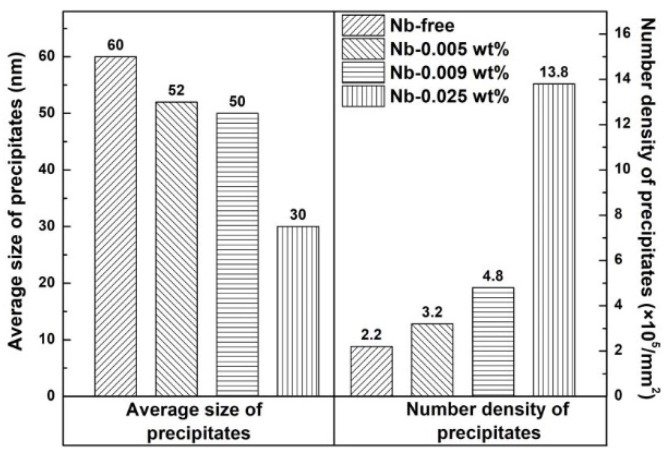
Size and number density of precipitates in hot-rolled bands.

In order to study the influence of the Nb content on precipitates in detail, the precipitate characteristics in each specimen was measured. Figure 2 shows the typical distribution of the precipitates in four samples, and the corresponding statistical results are shown in Figure 3. The average size and number density of precipitates in specimen S1 are 60 nm and 2.2 × 10^5^/mm^2^, respectively. In addition, the average size of the precipitate decreases and the number density of the precipitate increases after Nb is added into the silicon steel; the average size and number density of precipitates in specimen S4 are 30 nm and 13.8 × 10^5^/mm^2^, respectively. The result in Figure 3 shows that Nb plays an important role in the distribution of precipitates in hot-rolled grain-oriented silicon steel.

### 3.2. Microstructure and Texture of Hot-Rolled Bands with Different Nb Content

**Figure 4:**
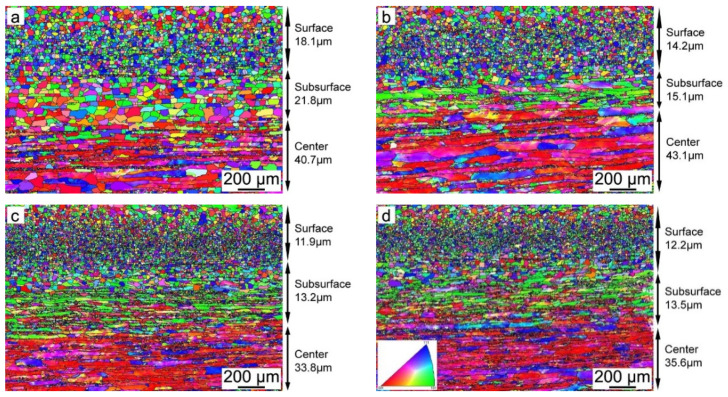
OIM of hot-rolled bands: (**a**) Nb-free, (**b**) Nb-0.005 wt%, (**c**) Nb-0.009 wt% and (**d**) Nb-0.025 wt%.

**Figure 5:**
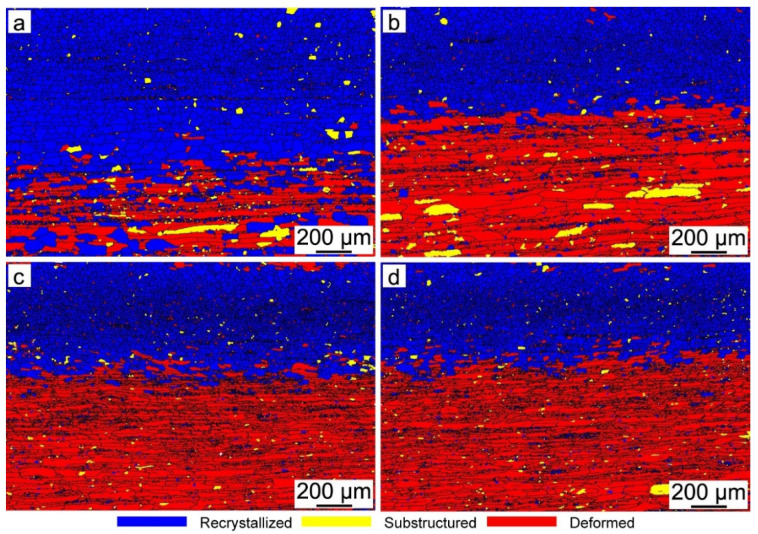
EBSD analysis of recrystallized microstructure of hot-rolled bands: (**a**) Nb-free, (**b**) Nb-0.005 wt%, (**c**) Nb-0.009 wt% and (**d**) Nb-0.025 wt%.

**Figure 6:**
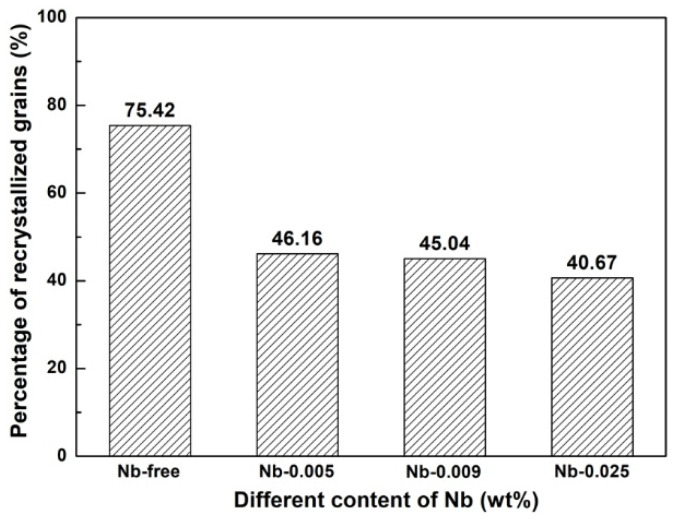
Percentage of recrystallized grains of hot-rolled bands with different Nb content.

Figure 4 shows the orientation imaging microscopy (OIM) of hot-rolled bands with different Nb content. It is obvious that the microstructure along the thickness is nonuniform, which is caused by the temperature gradient and the different amounts of deformation along the thickness. Fine equiaxed grains are observed in the surface regions, while complex microstructures of elongated grains and deformed structures are presented in the subsurface and center regions of the hot-rolled bands. Generally, the average grain size increases from the surface to the center for each sample, and the average grain size decreases after Nb is added into the silicon steel. Figure 5 shows the recrystallized microstructures of hot-rolled bands. The blue zone is the recrystallized structure, while the yellow zone and red zone are sub-structured and deformed structure, respectively. It can be observed that the percentage of recrystallized grains obviously decreases after Nb is added into silicon steel, as shown in Figure 6, while the amount of recrystallized grains in Nb-bearing hot-rolled bands shows almost the same fraction.

**Figure 7:**
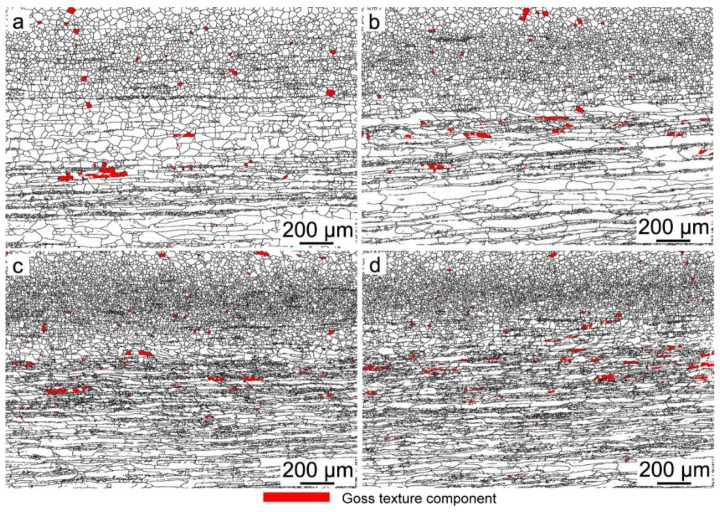
Goss texture of hot-rolled bands: (**a**) Nb-free, (**b**) Nb-0.005 wt%, (**c**) Nb-0.009 wt% and (**d**) Nb-0.025 wt%.

**Figure 8:**
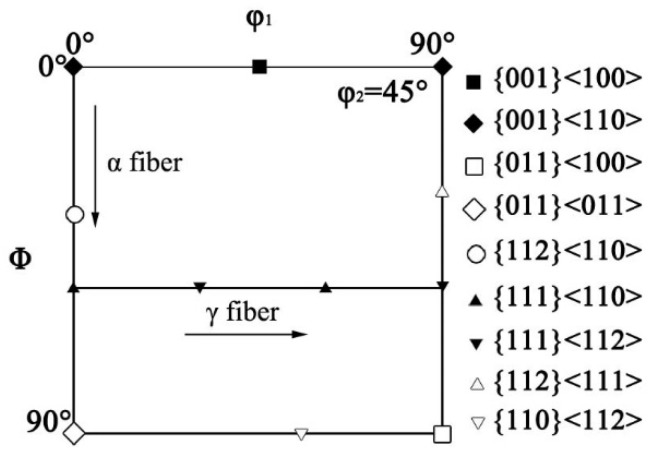
Ideal preferred orientations in Euler space.

**Figure 9:**
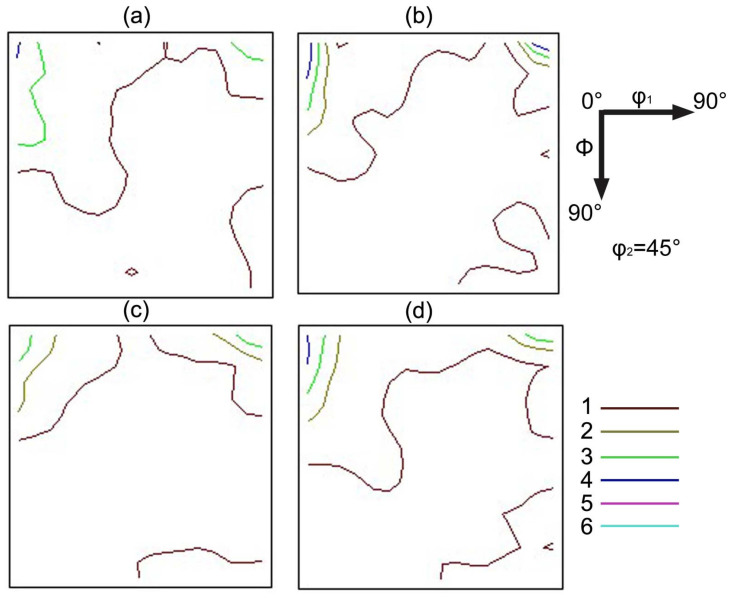
ODFs at φ_2_ = 45° of hot-rolled bands: (**a**) Nb-free, (**b**) Nb-0.005 wt%, (**c**) Nb-0.009 wt% and (**d**) Nb-0.025 wt%.

**Figure 10:**
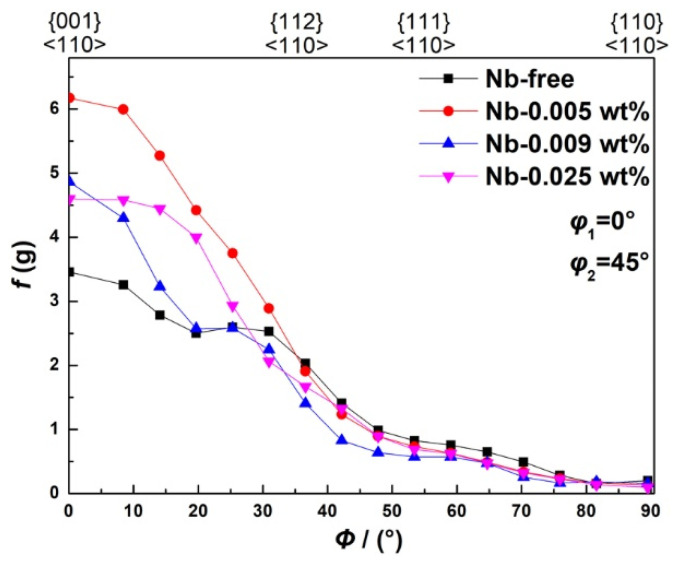
Orientation density distribution of α fiber.

**Figure 11:**
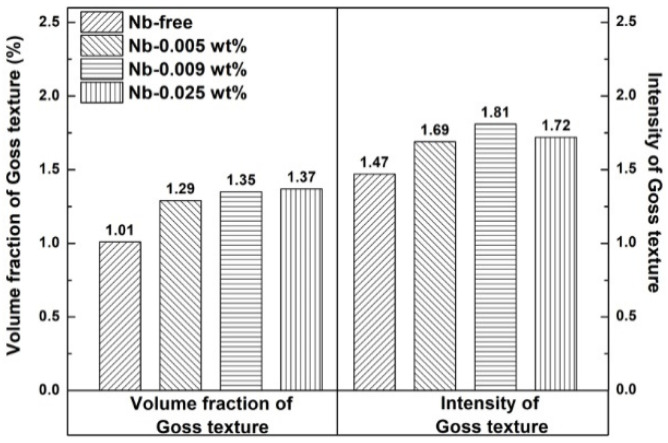
Proportion and intensity of Goss texture of hot-rolled bands with different Nb content.

Previous researchers have reported that the Goss texture is mainly located at the region around the surface of hot-rolled bands [25,26]; the distribution map of the Goss grains is shown in Figure 7, and the red zone is Goss texture within 10° of the exact Goss orientation. This indicates that the Goss grains are mainly located at the subsurface, and the volume fraction of the Goss texture in specimen S4 is the largest, at 1.37%. The ideal preferred orientations of cubic structure materials in the Euler space are located at the orientation distribution function (ODF) maps at φ_2_ = 45°, as shown in Figure 8. To further study the influence of Nb content on the texture of silicon steel, the graph of φ_2_ = 45° ODF at the region between the surface and the center of the plane vertical to TD in hot-rolled bands is shown in Figure 9. It can be observed that the textures in four hot-rolled bands are similar; the main components of the texture are {001}<110>, {112}<111> and {110}<001>. However, the intensity of α fiber firstly increases after Nb is added into silicon steel, while it decreases with the increase of the Nb content, as shown in Figure 10. The statistical Goss texture proportion and intensity in hot-rolled bands is shown in Figure 11. The addition of Nb plays a significant role on the Goss texture of the hot-rolled bands, and the larger amount and stronger intensity of the Goss texture are obtained in specimen S4.

## 4. Discussion

### 4.1. Effect of Nb on Precipitates in Hot-Rolled Bands

In this study, the main difference between the four samples is that the Nb content is different (0–0.025 wt%). Thus, niobium content is the main factor for different phenomena. It is reported that the inhibitors play a key role at the high temperature annealing stage of grain-oriented silicon steel and are mainly obtained during hot rolling [11]. It should be noticed that grain-oriented silicon steel has a dual phase (ferrite and austenite) at the temperature of 760–1200 °C. Both the solubility of AlN and Nb(C, N) in austenite and ferrite are different. Generally speaking, the dissolution temperature of AlN and Nb(C, N) in austenite are lower than those in ferrite [27,28]. Therefore, only considering that the solubility of AlN and Nb(C, N) in ferrite is not complete, the solubility of AlN and Nb(C, N) in austenite should be the main factor. The solubility products for MnS, AlN and Nb(C, N) in austenite can be calculated by following equations [28,29,30], where *T* is the temperature in Kelvin.

(1) $$\log[\mathrm{Mn}(\mathrm{wt}\%)\times S(\mathrm{wt}\%)]=-\frac{9220}{T}+2.929$$

(2) $$\log[\mathrm{Al}(\mathrm{wt}\%)\times N(\mathrm{wt}\%)]=-\frac{7400}{T}+1.95$$

(3) $$\log[\mathrm{Nb}(\mathrm{wt}\%)\times C(\mathrm{wt}\%)]=-\frac{7407}{T}+2.783$$

(4) $$\log[\mathrm{Nb}(\mathrm{wt}\%)\times N(\mathrm{wt}\%)]=-\frac{9940}{T}+3.82$$

According to the equations, the dissolution temperatures of MnS, AlN, NbC and NbN in different samples are listed in Table 2. It can be concluded that the precipitation order of the precipitates in the tested specimens is MnS > AlN > NbN > NbC. According to classical nucleation theory, the inhibitors that precipitate first will become the nucleus of the latter ones; thus, the newly formed AlN may take the preformed MnS as nuclei to reduce the Gibbs free energy, as shown in Figure 1a,b. When Nb is added into silicon steel, the composite precipitates of MnS and Nb(C, N) and composite precipitates of MnS, Nb(C, N) and AlN are formed through the same theory, as shown in Figure 1c–e. It should be observed that the dissolution temperatures of MnS or AlN in the four tested specimens are similar, while the dissolution temperatures of NbC and NbN increase with the increase of Nb. Before hot rolling, the ingots are reheated to 1180 °C for 90 min so the Nb(C, N) in tested specimens could completely dissolved into the matrix. Then, the C, N and Nb atoms start to form Nb(C, N) at dislocations caused by hot rolling with the decrease of temperature. Meanwhile, the precipitation start temperatures of NbC and NbN in specimen S4 are the highest, and the diffusion of atoms becomes faster at higher temperatures, which means this provides more time to precipitate for Nb(C, N) in specimen S4. When the temperature decreases to 940 °C, the hot-rolled bands were coiled at 580 °C by water-cooling immediately. There was no enough time to coarsen for the precipitates. As a result, finer and more dispersed precipitates were obtained in specimen S4, as shown in Figure 2, which is consistent with Feng’s results [16].

### 4.2. Effect of Precipitates on Microstructure and Texture of Hot-Rolled Bands

As is known to all, the shear deformation caused by the friction between the roller and strip surface declines gradually from the surface to the center during the rolling stage. Thus, the higher stored energy and higher dislocation density are obtained on the surface. Therefore, the heterogeneous microstructure along the thickness direction of the hot-rolled bands is obtained. The surface layer is full of fine, equiaxed and recrystallized grains, while a complex microstructure of elongated grains and deformed structures exists in the subsurface and center layer, as shown in Figure 4. In addition, it can be concluded from Figure 4, Figure 5 and Figure 6 that the average grain size and recrystallization fraction of hot-rolled bands is quite different after Nb is added into silicon steel. It is well known that static recrystallization occurs when the temperature is higher than above approximately half of the melting point (0.5 *T_m_*) for strain-hardened metals, and dynamic recrystallization can take place during straining, as long as the temperature is higher than 0.5 *T_m_* [31]. The grain boundary migration should be determined by the competition between the driving force for recrystallization and the pinning force of the small precipitates. The driving force and pinning force can be calculated by Equations (5) and (6) [32,33]:

(5) $$F_{R}=\frac{1}{2}\cdot \Delta \rho \cdot \mu \cdot b^{2}$$

(6) $$F_{P}=\frac{3}{2}\cdot \frac{\gamma \cdot F_{v}}{r}$$

where Δ*ρ* is the change in dislocation density associated with the migration of the recrystallization front into the deformed region, *µ* is the shear modulus, *b* is the Burgers vector, *γ* is grain boundary energy, *F_ν_* is the volume fraction of precipitates, *r* is the average precipitate radius, *F_R_* is the driving force for recrystallization and *F_P_* is the pinning force of the precipitates. It was proven that dynamic precipitation could hinder the dynamic recrystallization process but not prevent it when the pinning force was less than the driving force [34]. In this study, the tested specimens were treated in the same way; the driving force for the recrystallization of four specimens could be thought of as the same. It can be concluded that the pinning force of the precipitates is directly proportional to the volume fraction of the precipitates, and is inversely proportional to the average precipitate radius when the *γ* is the constant value. According to Section 4.1, with the increase of Nb content, the average size and number density of precipitates reduce and increase, respectively. Therefore, smaller grain sizes and less recrystallization fractions are presented in specimens with more dispersed, finer precipitates (specimen S4). However, it should be observed that the difference between the structures and recrystallization fractions of specimens S3 and S4 is rarely small, which indicates that the effect of the Nb addition is not obvious when the content is higher than 0.009 wt%.

The origin and development of Goss texture makes a significant influence on the successful production of grain-oriented silicon steel. It is reported that the ingots are hot rolled with a total reduction of over 90% in the conventional process, and the Goss texture originates from the shear texture [25,35,36]. It is observed in the present results (Figure 7) that the Goss grains are mainly located in the surface and subsurface layers. It is proven that the formation of the Goss texture component can be promoted by the fine precipitates, which can hinder the dynamic recrystallization during hot rolling [29]. In this study, finer and more dispersed precipitates were obtained in specimen S4, contributing to larger volume fractions of Goss texture in specimen S4, as shown in Figure 11. However, it should be observed that the intensity of the Goss texture in specimen S3 is much higher than that in specimen S4. Combining the two factors, it is reasonable to deduce that the effect of the Nb addition on texture is not obvious when the content is higher than 0.009 wt%.

## 5. Conclusions

In this study, four hot-rolled bands with different Nb content (0–0.025 wt%) were prepared. A detailed study of the precipitates, microstructure and texture of hot-rolled bands was carried out. The main conclusions are summarized as follows:

(1) The precipitates in hot-rolled bands were mainly single MnS and composite precipitates of MnS and AlN, and Nb(C, N) begins to precipitate after Nb is added. Meanwhile, with the increase of the Nb content, the precipitates become finer and more dispersed.
(2) The stronger pining force was obtained in the specimens with the Nb addition, which pinned up the deformed matrix and blocked the dynamic recrystallization during hot rolling; the structure and recrystallization fraction of the specimens with the Nb addition are finer and less, respectively. Larger volume fractions and a stronger intensity of Goss texture was formed in the specimens containing 0.025 wt% Nb, due to the finer and more dispersed precipitates.
(3) When the Nb content is higher than 0.009 wt%, the effect of the Nb addition on microstructure and texture is not obvious.

## Figures and Tables

**Table 1:** Chemical compositions of tested silicon steel (wt%).

Sample	C	Si	Mn	S	Al_s_	Nb	N	Fe
S1	0.056	3.14	0.110	0.0068	0.024	-	0.0076	Balance
S2	0.056	3.11	0.092	0.0064	0.026	0.005	0.0083	Balance
S3	0.057	3.13	0.091	0.0079	0.025	0.009	0.0082	Balance
S4	0.060	3.23	0.110	0.0064	0.026	0.025	0.0075	Balance

**Table 2:** Dissolution temperature of inhibitors/°C.

Inhibitors	AlN	MnS	NbN	NbC
S1	1027	1250	-	-
S2	1045	1223	939	896
S3	1039	1245	977	947
S4	1034	1243	1044	1048

## Data Availability

Not applicable.
